# Food Advertisements in Two Popular U.S. Parenting Magazines: Results of a Five-Year Analysis

**DOI:** 10.5539/gjhs.v6n2p175

**Published:** 2013-12-24

**Authors:** Corey H. Basch, Rodney N. Hammond, Danna Ethan, Lalitha Samuel

**Affiliations:** 1 Department of Public Health, William Paterson University, Wayne, NJ, USA; 2 Department of Health and Nutrition Sciences, Montclair State University, Montclair, NJ, USA; 3 Health Education and Promotion, Department of Health Sciences, Lehman College, The City University of New York, Bronx, NY, USA; 4 Dietetics, Foods, and Nutrition, Department of Health Sciences, Lehman College, The City University of New York, Bronx, NY, USA

**Keywords:** parenting magazines, food advertisements, obesity

## Abstract

Obesity rates among American youth have prompted an examination of food advertisements geared towards children. Research indicates children’s high exposure to these advertisements and their influence on food preferences. Less is known about the presence of these advertisements in parenting magazines. This study’s objective was to examine prevalence of food advertisements in popular parenting magazines and identify products by USDA food category. We analyzed 116 issues of two popular U.S. parenting magazines across five years. All food and beverage advertisements for USDA Food Category were coded. Breakfast cereals were coded for nutritional quality. The coding took place at varied libraries in New Jersey, in the United States. A total of 19,879 food and beverage products were analyzed. One-third of advertisements (32.5%) were for baked goods, snacks, and sweets -- products generally low in nutrient density. Two-thirds of the breakfast cereals were low in nutritional quality (64.6%). Beverages comprised 11% of the advertisements, fruit juices the highest proportion. Less than 3% of advertisements were for fruits and vegetables combined. No significant food product trends were evident across the five-year period. Food advertisements identified in parenting magazines were generally low in nutritional value. Additional research is necessary to determine the influence of food advertisements on parents’ purchasing habits.

## 1. Introduction

Over a third of American children and adolescents are categorized as overweight or obese (CDC, 2012a; Ogden et al., 2012; National Center for Health Statistics, 2012). The immediate consequences of childhood obesity include breathing and joint problems, gastro-esophageal reflux, social and psychological issues. Long-term consequences include risk of adulthood obesity with its related health effects (CDC, 2012b; Whitlock, Williams, Gold, Smith, & Shipman, 2005; Han, Lawlor, & Kimm, 2010; Swartz & Puhl, 2003; Taylor et al., 2006; Sutherland, 2008).

Children are eating less than the recommended allowance for whole grains, fruits and vegetables, and are consuming more high-carbohydrate and energy-dense food (Salinsky, 2006). Data from the National Health and Nutrition Examination Survey (NHANES) indicate that the three leading sources of energy for two- to eighteen-year-olds are grain desserts (e.g. cookies, cakes, donuts, granola bars), pizza, and soda (Reedy & Krebs-Smith, 2010). Concerning younger children, home-packed lunches of pre-schoolers have been shown to lack requisite servings of fruits, vegetables and milk (Sweitzer, Briley, & Robert-Gray, 2009). Adolescent snacking has increased by over 20% since the 1970s with a significant portion high in fat, added sugar, or both, leading to higher daily intake of calories (Sebastian, Goldman, & Enns, 2010).

A strong influence on food choices, various media channels including television, print, in-school marketing, movie cross-marketing, and web-based media show advertisements of sugar-sweetened beverages and foods high in fat, sugar, and sodium aimed at children (Sobal & Bisogni, 2009; Powell et al., 2011; Harrison & Marske, 2005; Story & French, 2004; Speers, Harris, & Schwartz, 2011). According to a recent Federal Trade Commission Report, the food and beverage industries spent 1.79 billion dollars in 2009 on advertising products to youth, using varied techniques to entice children who, in turn, influence their parents to purchase these foods (“pester power”) (Story & French, 2004; Ustjanauskas et al., 2010; Schwartz & Ustjanauska, 2012; Federal Trade Commission, 2012).

Literature on the effects of these advertisements (on television in particular) on children’s short-term food choices has grown in the past decade (Nestle, 2006; McGinnis, Gootman, & Kraak, 2006; Hasting, McDermott, Angus, Stead, & Thomson, 2006; Halford, Boyland, Hughes, Oliveira, & Dovey, 2007). Although an association between obesity in children and their exposure to food advertising has not been established (Salinsky, 2006), recent studies do suggest that children are more likely to request and choose foods that are advertised (Jones & Kervin, 2010; Arnos, 2006).

Evidence suggests that adults are also influenced by food advertisements (Harris, Bargh, & Brownell, 2009). Limited research suggests that food advertisements in parenting magazines do not reflect the United States Department of Agriculture’s (USDA) dietary guidelines for children (Manganello, Clegg Smith, Sudakow, & Summers, 2012). The USDA’s guidelines call for in-season fruits and vegetables, protein-rich foods, dairy products, and grains, with servings varying by age. The number of empty calories (those from added sugars and/or solid fats) should be limited depending on age, gender, and level of physical activity (USDA, 2011; USDA, 2013).

With these guidelines and the potential impact of children’s food advertisements on parents in mind, our study’s aim was to assess the type and prevalence of food and beverage advertisements, and nutritional quality for certain products advertised, in two popular parenting magazines. *Parents* and *Parenting* are widely read with a combined circulation of approximately 4 million and a combined readership estimated at over 20 million (GfK Mediamark Research and Intelligence, 2012).

## 2. Methods

### 2.1 Sample

Our sample consisted of 116 issues from two popular parenting magazines, *Parents* (n = 60) and *Parenting* (n = 56) from January 2007 to December 2011/January 2012. The sampling frame included all issues over this five-year period; in four cases, December and January issues were combined by the publisher. It should be noted that in February, 2009, parenting magazine split into two separate magazines. At this point we began coding Parenting Early Years. This study was deemed not human subjects research by the Institutional Review Boards at both William Paterson University and Lehman College.

### 2.2 Coding

A coding sheet was created using food categories from the USDA’s National Nutrient Database for Standard Reference (USDA, 2013b). From this highly detailed database, we selected food categories that were pertinent to the study, e.g. baked goods, sweets, snacks, and dairy products. For the USDA category of “Dairy,” we sought more specificity by creating subcategories including cheese, yogurt, and milk (1%, 2%, whole) to facilitate coding. For other USDA categories such as “snacks,” “sweets,” “baked products,” and “beverages” we included detailed food lists based on the database. To code and analyze nutritional quality of cereals, we referred to the Cereal FACTS Report developed by Yale University’s Rudd Center for Food Policy and Obesity (Cereal F.A.C.T.S, 2012). The report assigns children’s cereals a score based on total calories and mix of ingredients including fiber, sodium, and sugar (Cereal F.A.C.T.S, 2012b). Using the Cereal FACTS rankings by overall nutrition as a guide, we divided the list of 43 cereals in half, creating two discrete categories of ratings: “healthy” and “less healthy” (Cereal F.A.C.T.S, 2012b).

Page counts were taken for each magazine. We included all advertisements barring pull-out promotions as these were not considered magazine content. Buying guides or products endorsed by the magazine were not considered advertisements and therefore were not counted as such. Special circumstances such as multiple-page advertisements were counted as one advertisement.

### 2.3 Data Analysis

Prior to data collection, we pilot tested our instrument on 10 issues (5 of each magazine). The pilot testing consisted of creating and refining our coding sheet’s advertisement categories which we based on the frequency of ads shown in the magazines (appearing five or more times). For this study, the category of infant formula was excluded from coding as this was addressed in a prior paper (Basch, Shaffer, Hammond, & Rajan, 2013). The coding sheet was primarily based on a prior study of parenting magazines (Basch, Hillyer, & Basch, 2013) and was adapted for more in-depth coding of food advertisements. Magazines used in pilot testing were not included in our sample.

The total number of advertisements for each food category was calculated across the five-year study period. The proportion (%) of each food category relative to total number of food advertisements was also calculated (across five years and for each individual year). Numbers were compared to identify trends in food category representation over the five-year study period. All calculations were made using Microsoft Excel Version 12. Intra-rater reliability was assessed in a random selection of 10 percent (n = 12) of magazines. There was 99% accuracy in replicated results.

Descriptive statistics were used to describe the prevalence of products in each food group for each year of the study period. A One-Way Analysis of Variance (ANOVA) was conducted to identify differences in the prevalence of different food advertisements across the 5-year period. Chi-square statistics were done to compare the proportion of advertisements related to healthy and unhealthy foods during the study period. Independent t-tests were run to identify if there were significant changes in the trends of advertisements between healthy and less healthy foods.

## 3. Results

Table 1 summarizes proportion of food products advertised in *Parenting and Parenting* magazines from 2007 to 2012 coded by categories adapted from the USDA National Nutrient Database for Standard Reference. Of the total number of advertisements during this period (n = 19,879), 10.6% were food-based advertisements (n = 2101). One-third (32.5%, n = 682) were for baked food items (e.g. tortillas, crackers, waffles), snacks (e.g. beef jerky, potato chips, fruit rolls), and sweets (e.g. candy, ice cream, pudding). Non-alcoholic beverages (water, juice, cocoa and carbonated beverages including soda) accounted for over 11% of the food-based advertisements (n = 240). Among these, fruit juices accounted for over 68% (n = 164). For our study, we did not differentiate between unsweetened and sweetened fruit juices as the advertisements did not always clearly state this. Water and soda represented 9% (n = 22) and 10% (n = 24), respectively, of the non-alcoholic beverages.

**Table 1:** Proportion (%) of food products coded by categories adapted from The USDA National Nutrient Database for Standard Reference as advertised in *Parenting and Parenting* magazine from January 2007 to December 2011-January 2012

Food Category (N=2101)	Proportion of Food Advertisements (%, n)	
Baked food products	16.5%	(346)
Soups and sauces	10.2%	(215)
Sweets	9.3%	(195)
Juice	7.8%	(164)
Snacks	6.7%	(141)
Meals and entrees	6.6%	(139)
Milk	6.2%	(131)
Cereal grains and pasta	5.8%	(121)
Spices and herbs	3.6%	(76)
Poultry products	3.3%	(70)
Beef products	3.2%	(67)
Breakfast cereals, unhealthy	2.5%	(53)
Baby foods	2.0%	(42)
Fruits	1.8%	(38)
Nuts and seeds	1.6%	(33)
Yogurt	1.6%	(33)
Breakfast cereals, healthy	1.4%	(29)
Cocoa	1.4%	(30)
Soda	1.1%	(24)
Pork products	1.1%	(23)
Cheese	1.0%	(21)
Vegetables	0.8%	(16)
Alcohol	0.7%	(15)
Lamb, game and legume products	0.5%	(11)
Restaurant foods	0.2%	(4)
Other dairy products	0.1%	(2)

Dairy products comprised 9% of the food advertisements and included milk (6.2%, n = 131), yogurt (1.6%, n = 33), and cheese (1.0%, n = 21). Another 8% represented meat including poultry (3.3%, n = 70), beef (3.2%, n = 67), and pork (1.1%, n = 23).

Prepared foods such as meals, entrees, and restaurant foods accounted for almost 7% of the food advertisements (n = 143). Soups and sauces also made a significant contribution representing over 10% (n = 215) of the foods advertised. Fewer than 3% of advertisements were for fresh fruits and vegetables (n = 54). These included 38 advertisements for fruits and 16 for vegetables. Only 1.6% were devoted to nuts and seeds (n = 33).

Breakfast cereals accounted for less than 4% of the food-related advertisements (n = 82) with almost two-thirds (64.6%, n = 53) categorized as “less healthy.” Cereals coded as “less healthy” were higher in sugar and sodium and lower in fiber than those coded as “healthy.”

Table 2 represents trends in annual representation of different food categories as a proportion (%) of the total food advertisements for each year. Although the combined representation of fruits, vegetables, nuts, and seeds more than doubled to 4.9% in 2010 and 7.5% in 2011 in comparison with 1.2% in 2007, the increases were meager as compared with baked goods which plateaued at more than one-third of the advertised foods. Among other food categories, there were no significant trends across the five-year study period. A one-way ANOVA confirmed no significant difference in the sale of different products over the study period (p>0.05).

**Table 2:** Proportion (%) of annual representation for different food product categories advertised in *Parenting and Parenting Early Years* magazines from January 2007 to December 2011-January 2012

Combined Food Categories	Annual Proportion (%) of Food Advertisements For Each Year				
	2007(N= 511)	2008(N=474)	2009(N=384)	2010(N=386)	2011(N=346)
Baked products, snacks and sweets	35.8	28.3	26.0	36.0	36.4
Breakfast cereals, grains and pasta	8.8	10.5	6.3	13.5	9.2
Fruits, vegetables, nuts and seeds	1.2	1.5	7.6	4.9	7.5
Dairy	9.6	10.5	7.8	8.3	7.5
Meat and legumes	2.2	10.8	12.0	8.3	9.0
Meals/entrees and restaurant foods	7.6	11.2	8.9	1.6	3.2
Soups and sauces	10.4	7.6	11.7	11.1	11.0
Spices and herbs	2.7	5.9	5.5	1.8	1.7
Water	1.4	0.6	1.6	1.6	0.0
Non-alcoholic beverages (except water), includes juice and soda	10.2	7.2	8.3	8.8	10.4
Alcohol	1.2	1.3	0.0	0.5	0.3

When the products advertised were categorized as “healthy” and “less healthy,” the prevalence of advertisements devoted to “less healthy” foods (such as unhealthy breakfast cereals, baked products, snacks, restaurant foods, meals and entrees, juices, sodas and meats) was substantially greater than the proportion of advertisements geared towards the healthier options such as fruits, vegetables, dairy products, healthy breakfast cereals, nuts and seeds. However, results of a t-test indicated no statistically significant difference in the trends of advertisements for healthy and less healthy foods during the study period (p > 0.05).

## 4. Discussion

This study’s findings are noteworthy for several reasons. Baked foods, snacks, and sweets accounted for the highest percentage of advertisements. These products are generally refined, high in added sugars and fats, and low in fiber. Fruit and vegetable advertisements were disproportionately low despite these food choices being excellent, low-calorie sources of fiber, vitamins, and phytonutrients. In a study of advertisements in six parenting magazines across four months, similar findings were noted (Manganello et al., 2012). Soups and sauces were the third highest food category advertised to parents, according to our data. The CDC has ranked soups fifth among ten leading sources of sodium (CDC, 2012c). According to Yang et al. (2012), high blood pressure risk among children is associated with sodium intake. This risk is even greater for obese youth.

There was an inadequate representation for products rich in vitamin D and calcium, nutrients essential for optimal bone growth in children. Observational studies suggest that replacing milk with carbonated drinks high in phosphates may be associated with decreased bone mass and increased risks for fractures (Calvo, 1993; Heaney & Rafferty, 2001). It is vital that the elevated nutritional requirement for calcium and vitamin D are fulfilled, particularly during puberty, in order to attain bone mass and prevent early onset of osteoporosis (Mesias, Seiquer, & Navarro, 2011).

We also observed few advertisements for breakfast cereals; of those, significantly more were coded as “less healthy.” Eating a healthy breakfast is critical for optimal nutrition in childhood. Research suggests that not eating a healthful breakfast can result in inadequate nutrient intake that may not be compensated for throughout the day (Nicklas & O’Neil, 2004). A study by Schwartz, Vartanian, Wharton and Brownell (2008) reported that 66% of children’s cereals did not meet the national nutrition standards. Our findings are similar showing that, across the five-year period, 64.6% of the advertised breakfast cereals were identified as “less healthy” suggesting an urgency for marketing practices to be redirected towards cereals of better nutritional quality (Cereal F.A.C.T.S., 2012b).

We noted no significant trends in food advertising over the five-year period. This finding should be examined in the context of the childhood obesity epidemic. Public health efforts at the national, state, and local levels to address this problem have been numerous and include raising public awareness of the importance of a nutritious diet. Examples are healthier food options in school cafeterias and widespread efforts like First Lady Michelle Obama’s *Let’s Move* campaign. Local efforts are exhibiting positive results as well. New York City’s Green Cart Initiative, improved vending machine standards, and chain restaurant calorie counts have been well-received as measures to reduce obesity (The New York City Obesity Task Force, 2012). The lack of significant advertising trends towards more healthful options in parenting magazines suggests that companies marketing food products through this medium are not readily joining this effort to reduce childhood obesity. This is especially troubling given the aims of these magazines to provide “honest, real-world advice,” “impact the growth and development of future generations,” and “advocate continuously and tirelessly for children” (Bonnier Parenting Media Kit, 2013; Parenting Media Kit, 2013; Parents Media Kit, 2013).

This study’s limitations include a small sampling frame and excluding other magazines in circulation that cover parenting content. Additionally, although the data collection instrument was developed by the team of authors, only one was responsible for coding the food products. Finally, our study included five years’ worth of magazines; had we included a ten-year sample, significant trends may have been noted.

Despite these limitations, our study addresses an important gap in current literature. Using the National Library of Medicine, we used “parenting,” “parents,” “magazines,” and “food advertisements” as key terms to identify papers related to food advertisements and parenting magazines. We then searched reference lists in the identified papers to further identify other relevant studies. We identified no other studies that analyzed multi-year trends in food advertisements in parenting magazines. Because the circulation of the magazines we analyzed is over four million (GfK Mediamark Research and Intelligence, 2012), we can assume that many parents, presumably prescribing to these magazines for months, or even years, are exposed to these food advertisements. How these advertisements affect parents’ purchasing behavior is unclear in the public health literature, but one qualitative study noted that parents were unaware of tactics used in food marketing. Nor were they aware of the deleterious effects these ads may have on children (Ustjanauskas et al., 2010).

This study offers insight into the prevalence and type of food and beverage advertisements found in two popular U.S. based parenting magazines. Future studies are necessary to determine the impact these advertisements have on parents’ purchasing behaviors. Practically, this is an opportune time for nutrition educators to consider developing and implementing health education interventions that highlight marketing strategies of advertisers. Given high readership of these magazines and consequent exposure to food and beverage advertisements, particularly for products like baked goods, snacks and sweets, it is essential to provide parents accurate information and strategies to become educated consumers. Aiding parents in understanding the nutritional value of foods typically advertised and promoting self-efficacy in making healthful food purchases for their families is of critical importance.
